# Supplementary material for: Compounding future escalation of emissions- and irrigation-induced increases in humid-heat stress
Source: Nat Commun. 2025 Oct 22;16:9326. doi: 10.1038/s41467-025-64375-1 (PMC12546594; doi:10.1038/s41467-025-64375-1)
Supplement: Supplementary file 1 — Supplementary Information [file 41467_2025_64375_MOESM1_ESM.pdf]

Supplementary Information - Compounding future escalation of emissions- and irrigation-induced increases in humid-heat stress

Yao et al.

Contents

List of Tables 3

List of Figures 4

1 Supplementary Tables 6

1.1 Supplementary Tables 6

2 Supplementary Figures 8

2.1 Supplementary Figures 1: Appendix Figures 8

2.2 Supplementary Figures 2: Dataset generated for SSP5-8.5 16

Supplementary References 23

List of Tables

S1 **Three groups of IPCC reference regions.** 13 IPCC regions are categorised into three groups for analysis. 6

S2 **Correlation between different socio-economic or hydro-climatic variables and current national irrigation techniques share** Spearman's coefficiency between present-day irrigation techniques fraction from the present-day irrigation techniques fraction[1] and socio-economic (GDP: gross domestic product, GOV: governance, URB: urbanisation, and GII: gender inequality index) or hydro-climatic (P: precipitation, PPET: precipitation divided by potential evapotranspiration, and TWS: terristrial water storage) variables (* indicates *p value < 0.1*, ** indicates *p value < 0.5*, *** indicates *p value < 0.01*) 6

S3 **Reference irrigation techniques change from USA and Iran.** Fraction of different irrigation techniques in the USA and Iran at the national level during the historical period. The trend is calculated with the lineal regression. 6

S4 **Suitable irrigation techniques for different crop functional types.** . . . 7

S5 **Adjusting factors based on socio-economic capacity and hydro-climatic complexity.** Based on the correlation between the variables and present-day irrigation techniques fraction (Table S2), these factors are assumed to project future irrigation techniques share. 7

List of Figures

S1 Fraction of sprinkler irrigation in the year 2010 (**a-b**), 2035 (**c-d**), 2070 (**e-f**), and 2100 (**g-h**), under SSP1-2.6 and SSP3-7.0. The spatial coverage of the IPCC reference regions[2] used in this study is indicated by solid lines, dashed lines, and dash-dotted lines. 9

S2 Fraction of drip irrigation in the year 2010 (**a-b**), 2035 (**c-d**), 2070 (**e-f**), and 2100 (**g-h**), under SSP1-2.6 and SSP3-7.0. The spatial coverage of the IPCC reference regions[2] used in this study is indicated by solid lines, dashed lines, and dash-dotted lines. 10

S3 Group 1's regional areas equipped for irrigation (AEI) equipped with different irrigation techniques under SSP1-2.6 (**left column**)and SSP3-7.0 (**right column**). Regions include Central North America (**a,b**), East Asia (**c,d**), and West North America (**e,f**). 11

S4 Group 2's regional areas equipped for irrigation (AEI) equipped with different irrigation techniques under SSP1-2.6 (**left column**)and SSP3-7.0 (**right column**). Regions include Mediterranean (**a,b**), West Central Asia (**c,d**), Arabian Peninsula (**e,f**), South Asia (**g,h**), and Southeast Asia (**i,j**). 12

S5 Group 3's regional areas equipped for irrigation (AEI) equipped with different irrigation techniques under SSP1-2.6 (**left column**)and SSP3-7.0 (**right column**). Regions include Southwest South America (**a,b**), Sahara (**c,d**), West Africa (**e,f**), West South Africa (**g,h**), and East South Africa (**i,j**). 13

S6 Regional time series of annual irrigation water withdrawal 14

S7 Traditional and new irrigation hot spot grid cells 14

S8 Illustrative flowchard for irrigation techniques share change 15

S9 Map of flood irrigation fraction 16

S10 Map of flood irrigation fraction 17

S11 Fraction of sprinkler irrigation in the year 2010 (**a**), 2035 (**b**), 2070 (**c**), and 2100 (**d**), under SSP5-8.5. The spatial coverage of the IPCC reference regions[2] used in this study is indicated by solid lines, dashed lines, and dash-dotted lines. . . . 18

S12 Fraction of drip irrigation in the year 2010 (**a**), 2035 (**b**), 2070 (**c**), and 2100 (**d**), under SSP5-8.5. The spatial coverage of the IPCC reference regions[2] used in this study is indicated by solid lines, dashed lines, and dash-dotted lines. 19

S13 Group 1's regional areas equipped for irrigation (AEI) equipped with different irrigation techniques under SSP5-8.5. Regions include Central North America (**a**), East Asia (**b**), and West North America (**c**). 20

S14 Group 2's regional areas equipped for irrigation (AEI) equipped with different irrigation techniques under SSP5-8.5. Regions include Mediterranean (**a**), West Central Asia (**b**), Arabian Peninsula (**c**), South Asia (**d**), and Southeast Asia (**e**). 21

S15 Group 3's regional areas equipped for irrigation (AEI) equipped with different irrigation techniques under SSP5-8.5. Regions include Southwest South America (**a**), Sahara (**b**), West Africa (**c**), West South Africa (**d**), and East South Africa (**e**). 22

1 Supplementary Tables

1.1 Supplementary Tables

Table S1: **Three groups of IPCC reference regions.** 13 IPCC regions are categorised into three groups for analysis.

Group	Regions
1	West North America, Central North America, East Asia
2	Mediterranean, West Central Asia, Arabian-Peninsula, South Asia, Southeast Asia
3	Southwest South America, Sahara, West Africa, West South Africa, East South Africa

Table S2: **Correlation between different socio-economic or hydro-climatic variables and current national irrigation techniques share** Spearman's coefficiency between present-day irrigation techniques fraction from the present-day irrigation techniques fraction[1] and socio-economic (GDP: gross domestic product, GOV: governance, URB: urbanisation, and GII: gender inequality index) or hydro-climatic (P: precipitation, PPET: precipitation divided by potential evapotranspiration, and TWS: terristrial water storage) variables (* indicates *p value < 0.1*, ** indicates *p value < 0.5*, *** indicates *p value < 0.01*)

	Flood fraction	Sprinkler Fraction	Drip Fraction
GDP	-0.5252***	0.4803***	0.4155***
GOV	-0.5302***	0.4625***	0.4676***
URB	-0.4428***	0.3796***	0.3723***
GII	0.4542***	-0.4104***	-0.3063***
P	0.2709***	-0.2110**	-0.2483***
PPET	-0.0203	0.0761*	-0.1773**
TWS	0.2082**	-0.1353	-0.3112***

Table S3: **Reference irrigation techniques change from USA and Iran.** Fraction of different irrigation techniques in the USA and Iran at the national level during the historical period. The trend is calculated with the lineal regression.

USA	1985	1990	1995	2000	2005	2010	2015
flood (%)	61.18	60.31	54.91	47.51	43.53	42.03	36.77
sprinkler (%)	38.82	39.69	42.23	45.74	49.84	50.58	54.58
drip (%)	0	0	2.86	6.75	6.63	7.39	8.65
	Trend in flood irrigation: -0.87% / year						
Iran	2003	2009	2013	2015	2019		
flood (%)	94.41	91.61	85.16	83.71	76.15		
sprinkler (%)	3.60	5.29	8.55	9.36	11.85		
drip (%)	1.99	3.10	6.29	6.93	12.00		
	Trend of flood irrigation: -1.21% / year						

Table S4: **Suitable irrigation techniques for different crop functional types.**

Crop type	drip	sprinkler	flood
Spring wheat	No	Yes	Yes
Winter wheat	No	Yes	Yes
Barley	No	Yes	Yes
Winter barley	No	Yes	Yes
Rye	No	Yes	Yes
Winter rye	No	Yes	Yes
Rice	No	No	Yes
Temperate maize	No	Yes	Yes
Tropical maize	No	Yes	Yes
Millet	No	Yes	Yes
Sorghum	No	Yes	Yes
Pulses	Yes	Yes	Yes
Cassava	No	No	No
Sunflower	Yes	Yes	Yes
Temperate soybean	Yes	Yes	Yes
Tropical soybean	Yes	Yes	Yes
Groundnuts	No	Yes	Yes
Rapeseed	No	Yes	Yes
Sugarcane	No	Yes	Yes
Citrus	Yes	Yes	Yes
Cocoa	Yes	Yes	Yes
Coffee	Yes	Yes	Yes
Cotton	Yes	Yes	Yes
Date palm	Yes	Yes	Yes
Grapes	Yes	Yes	Yes
Oil palm	Yes	Yes	Yes
Potatoes	Yes	Yes	Yes
Fodder grass	No	Yes	Yes
Miscanthus	No	Yes	Yes

Table S5: **Adjusting factors based on socio-economic capacity and hydro-climatic complexity.** Based on the correlation between the variables and present-day irrigation techniques fraction (Table S2), these factors are assumed to project future irrigation techniques share.

Socio-economic capacity	<0.5	0.5-0.75	0.75-1	1-1.25	>1.25
	-0.8	-0.4	0	+0.4	+0.8
Hydro-climatic complexity	<0.25	0.25-0.5	0.5-0.75	0.75-1	>1
	-0.2	-0.1	0	+0.1	+0.2

2 Supplementary Figures

2.1 Supplementary Figures 1: Appendix Figures

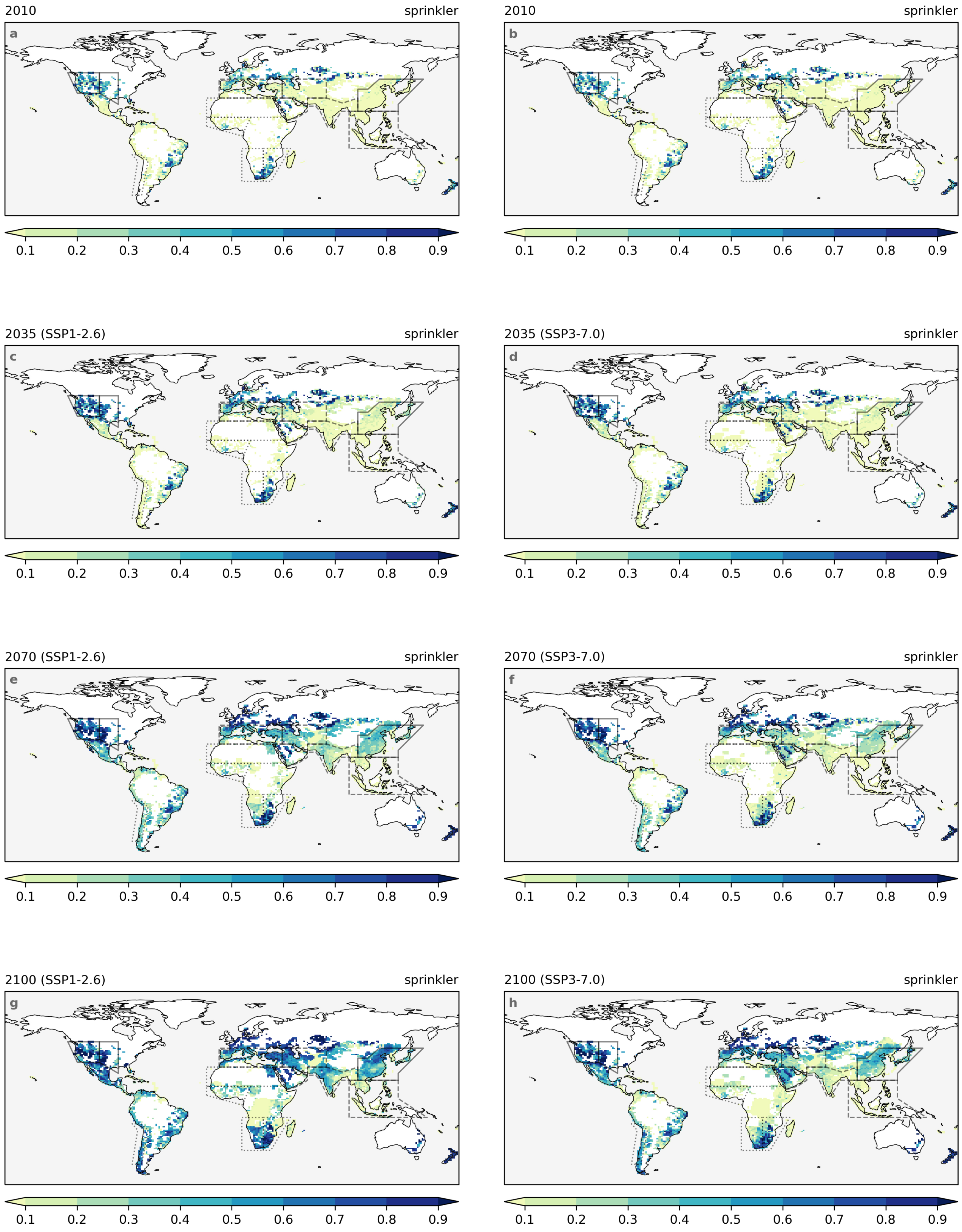

Supplementary Figure S1 | Fraction of sprinkler irrigation in the year 2010 (**a-b**), 2035 (**c-d**), 2070 (**e-f**), and 2100 (**g-h**), under SSP1-2.6 and SSP3-7.0. The spatial coverage of the IPCC reference regions[2] used in this study is indicated by solid lines, dashed lines, and dash-dotted lines.

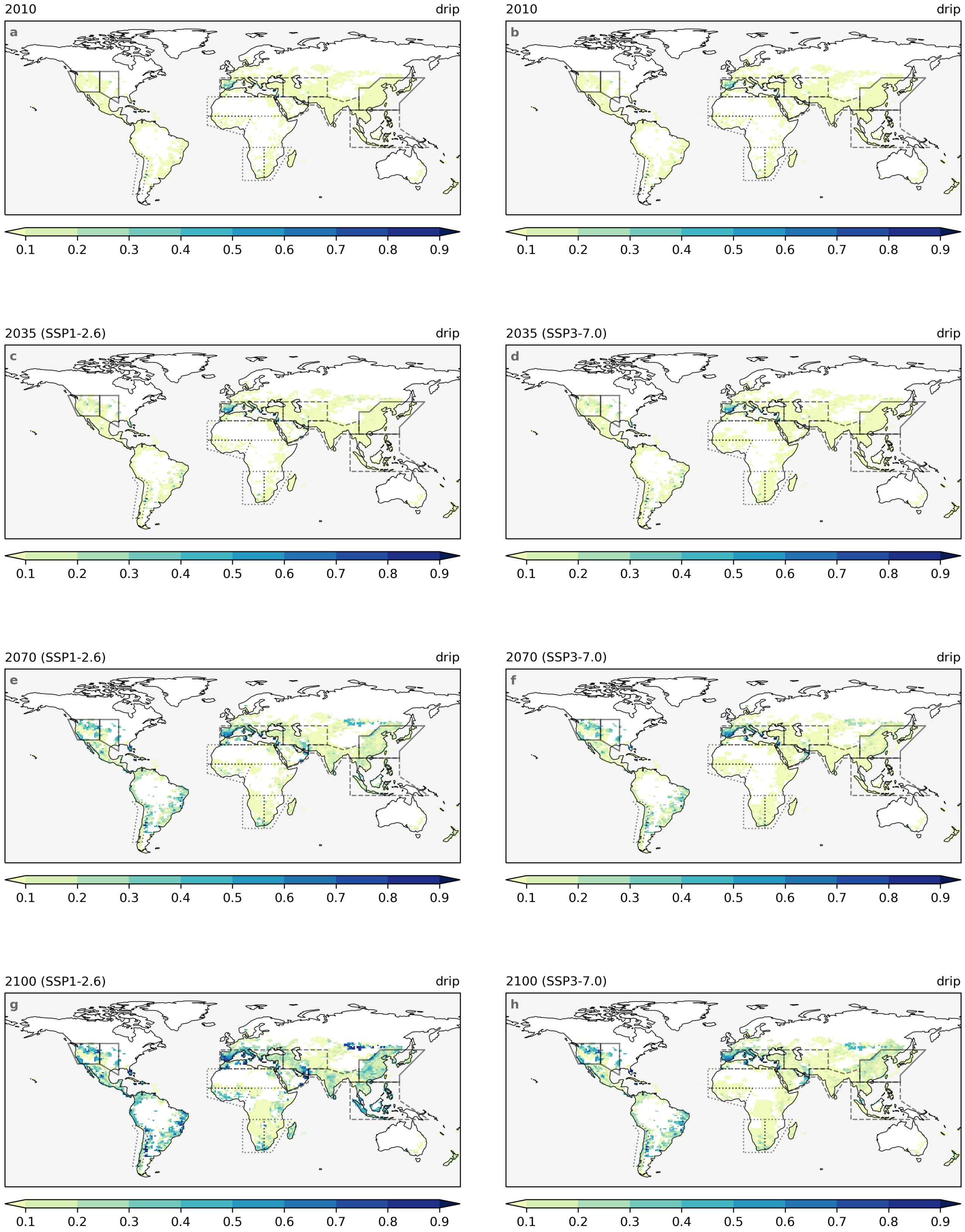

Supplementary Figure S2 | Fraction of drip irrigation in the year 2010 (**a-b**), 2035 (**c-d**), 2070 (**e-f**), and 2100 (**g-h**), under SSP1-2.6 and SSP3-7.0. The spatial coverage of the IPCC reference regions[2] used in this study is indicated by solid lines, dashed lines, and dash-dotted lines.

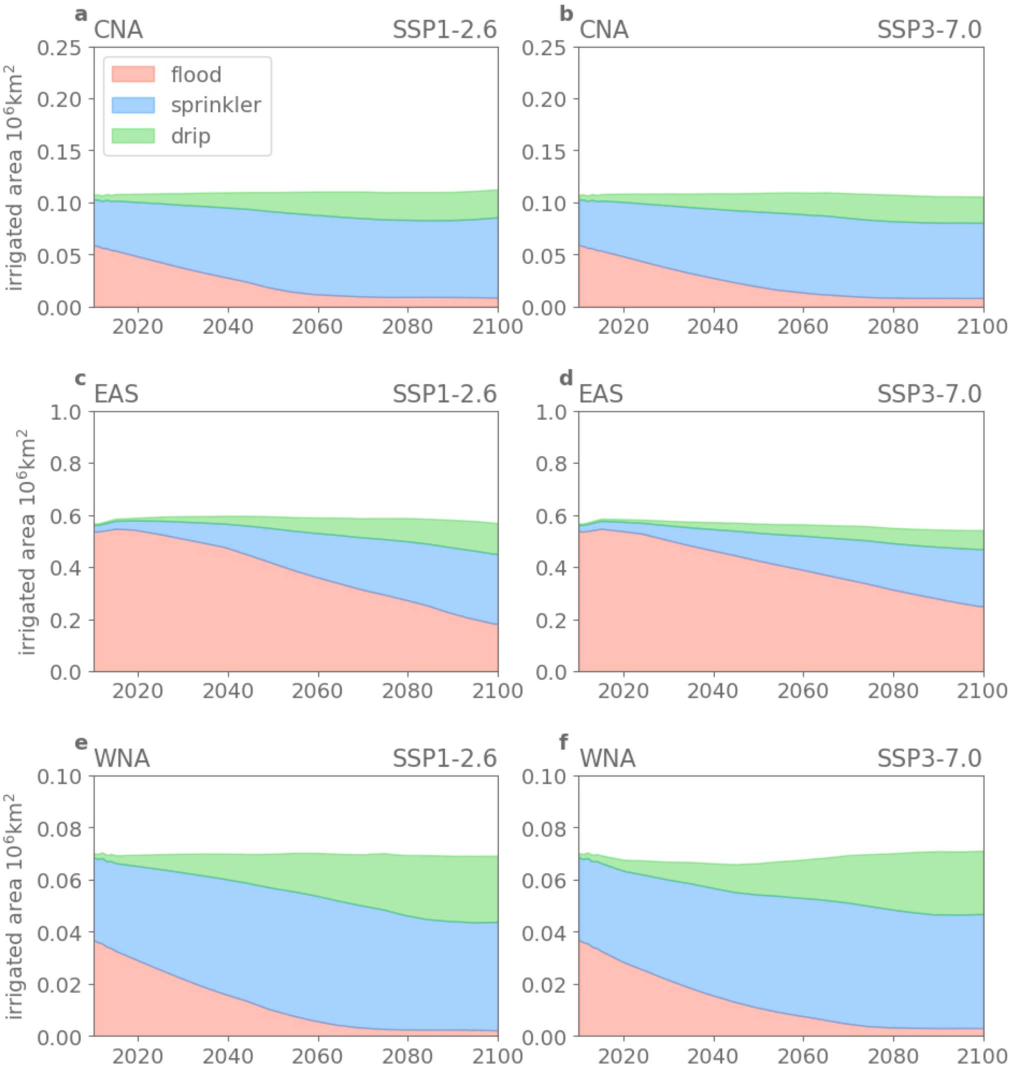

Supplementary Figure S3 | Group 1's regional areas equipped for irrigation (AEI) equipped with different irrigation techniques under SSP1-2.6 (**left column**)and SSP3-7.0 (**right column**). Regions include Central North America (**a,b**), East Asia (**c,d**), and West North America (**e,f**).

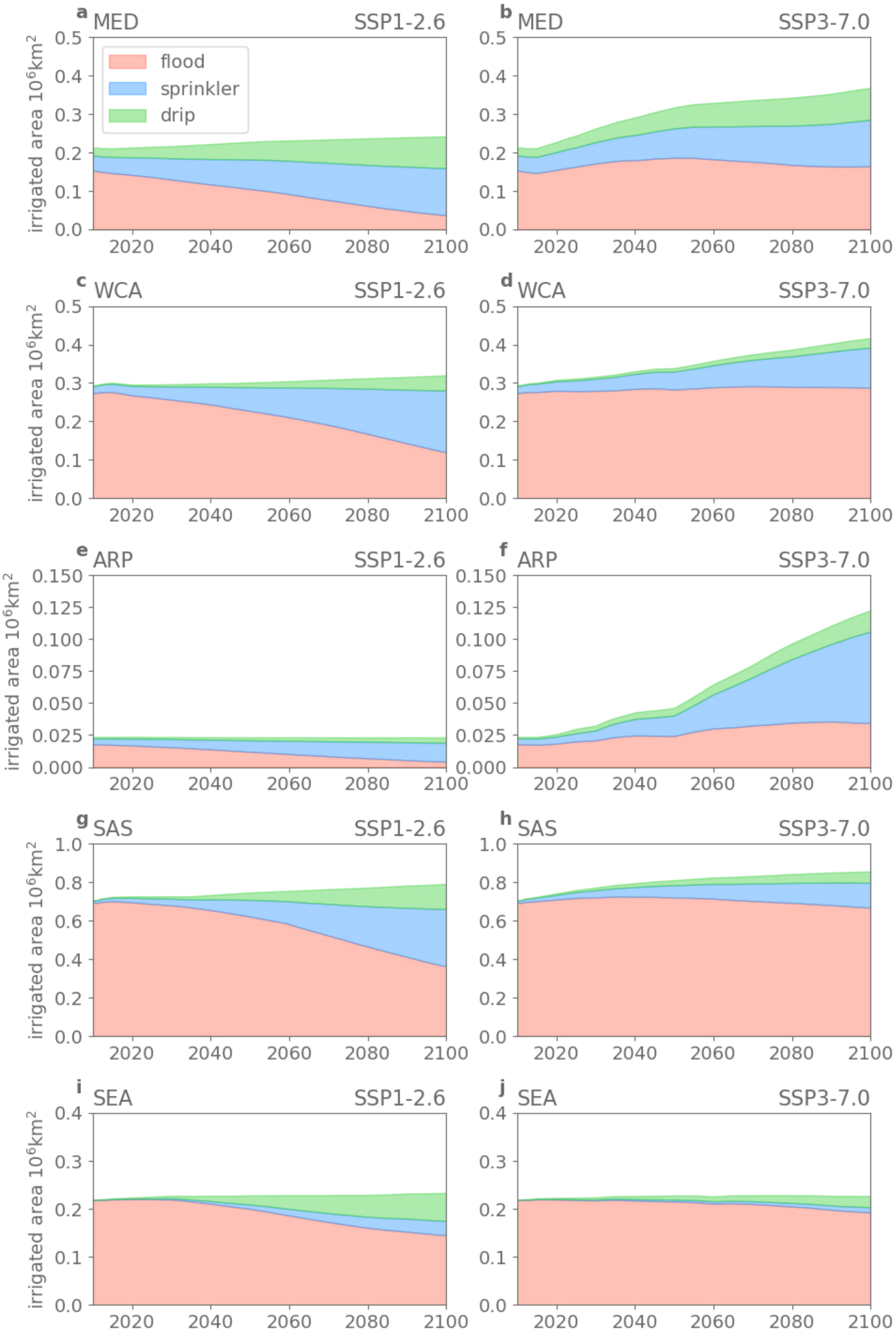

Supplementary Figure S4 | Group 2's regional areas equipped for irrigation (AEI) equipped with different irrigation techniques under SSP1-2.6 (**left column**)and SSP3-7.0 (**right column**). Regions include Mediterranean (**a,b**), West Central Asia (**c,d**), Arabian Peninsula (**e,f**), South Asia (**g,h**), and Southeast Asia (**i,j**).

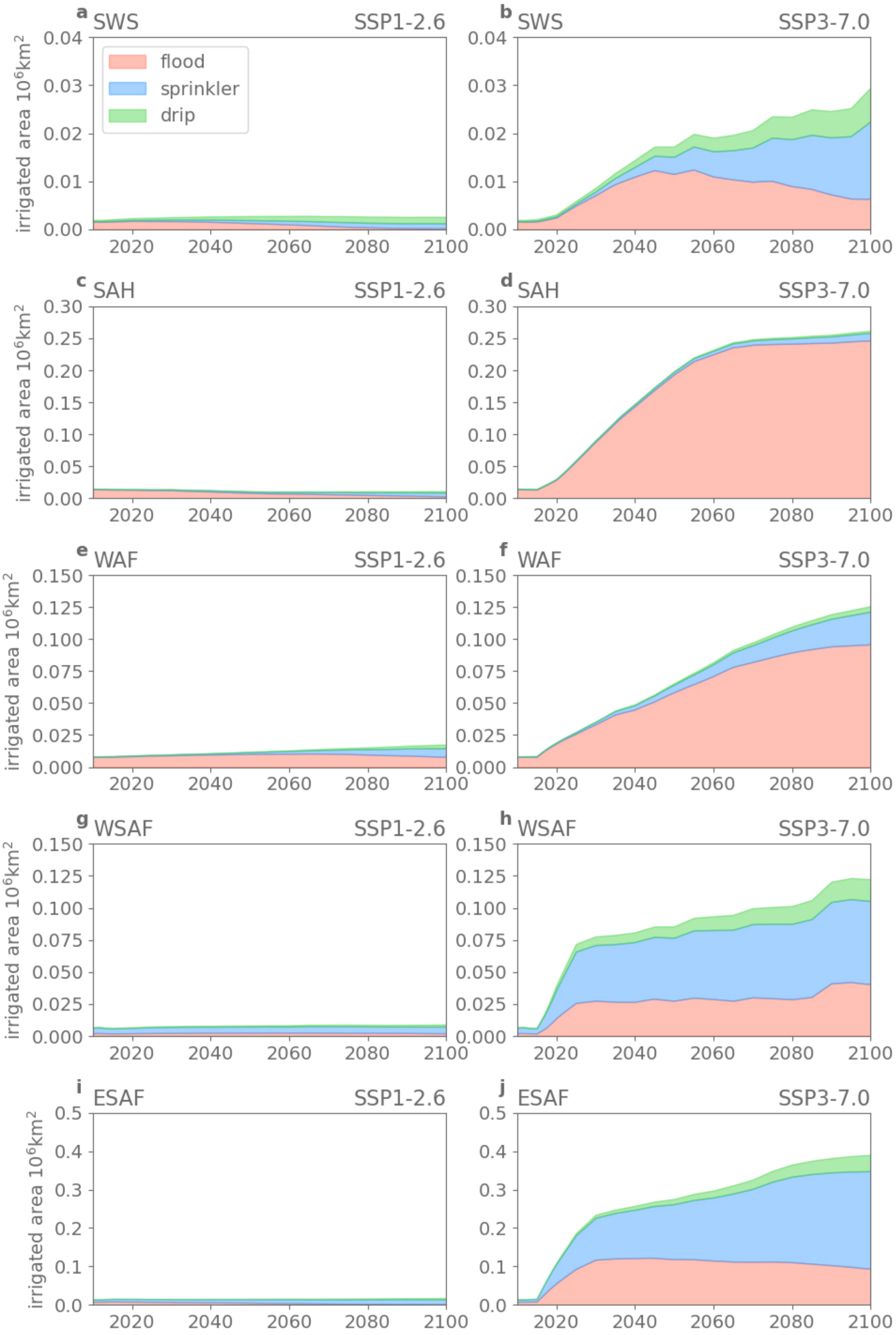

Supplementary Figure S5 | Group 3's regional areas equipped for irrigation (AEI) equipped with different irrigation techniques under SSP1-2.6 (**left column**)and SSP3-7.0 (**right column**). Regions include Southwest South America (**a,b**), Sahara (**c,d**), West Africa (**e,f**), West South Africa (**g,h**), and East South Africa (**i,j**).

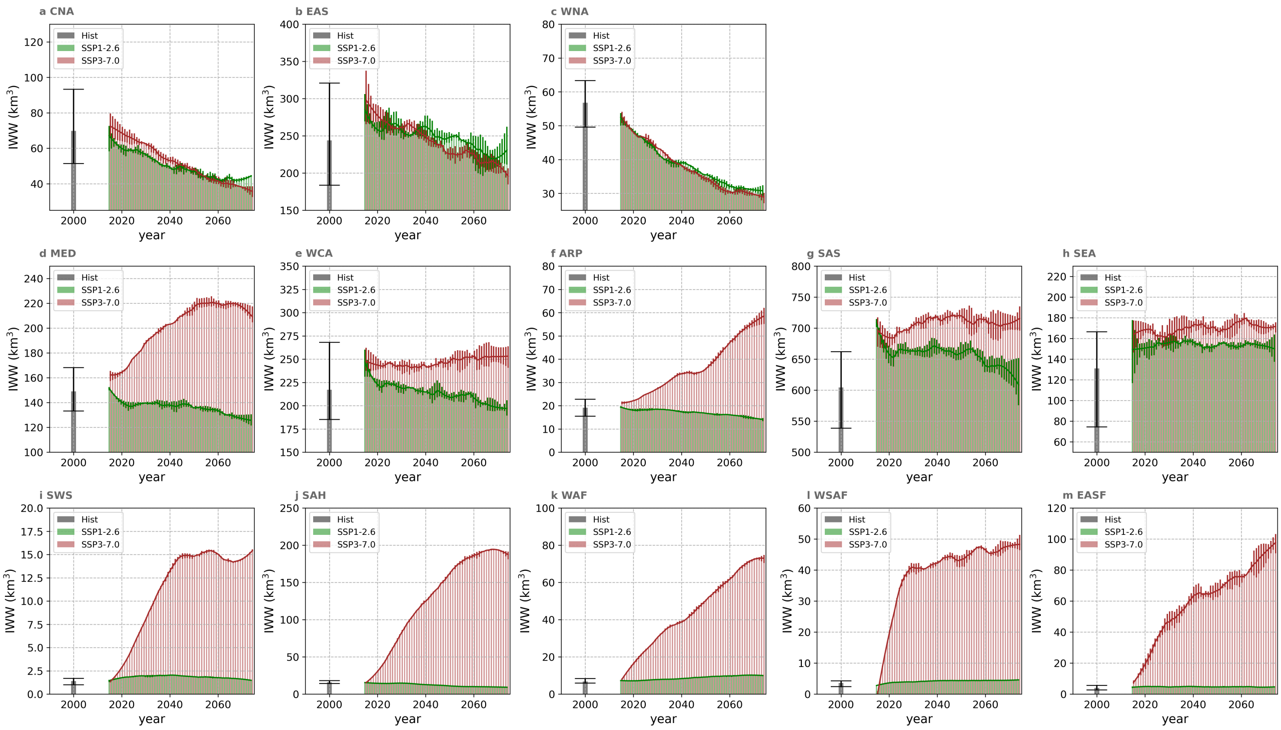

Supplementary Figure S6 | Regional summed irrigation water withdrawal of individual regions.

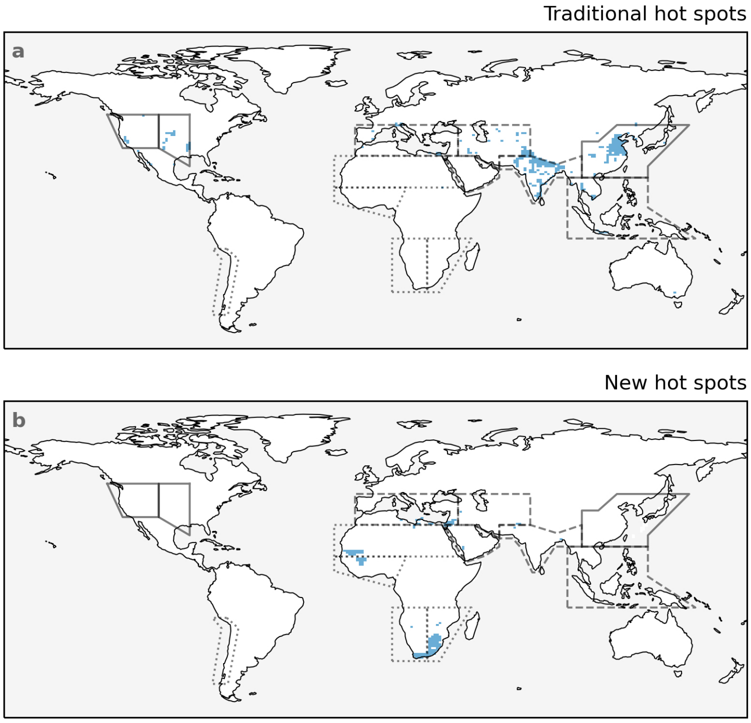

Supplementary Figure S7 | Traditional and new irrigation hot spot grid cells. a Grid cells with more than 20% of irrigated area in the year 2010. **b** Grid cells with less than 20% of irrigated area in the year 2010 but more than 20% in the year 2070 under SSP3-7.0. The spatial coverage of the IPCC reference regions[2] used in this study is indicated by solid lines, dashed lines, and dash-dotted lines.

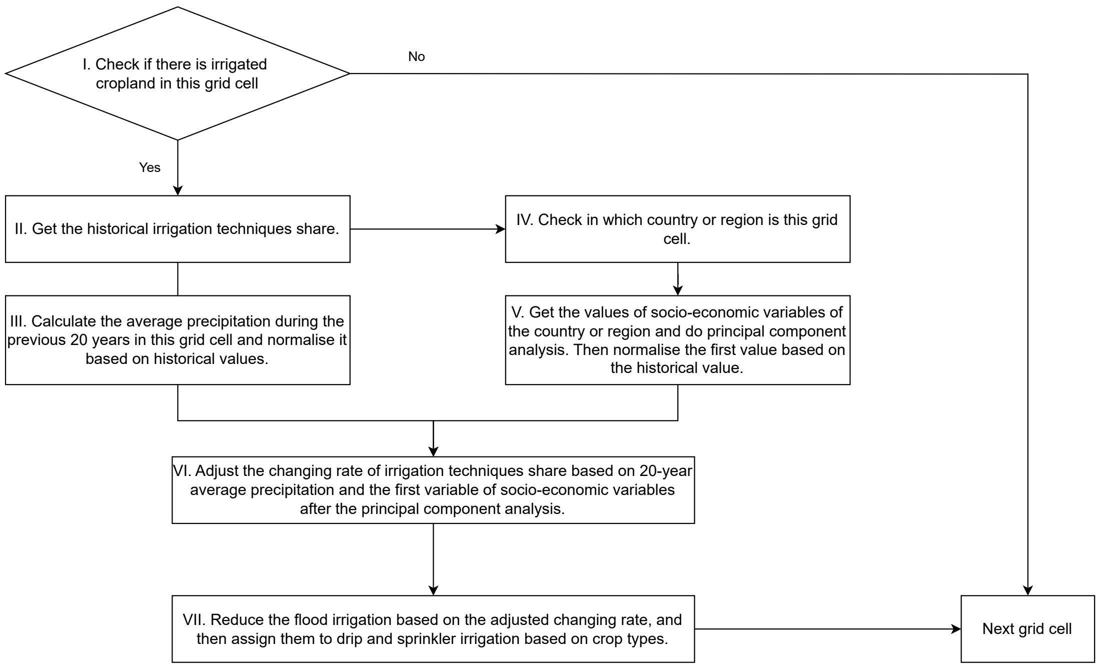

Supplementary Figure S8 | Illustrative flowchard for irrigation techniques share change.

2.2 Supplementary Figures 2: Dataset generated for SSP5-8.5

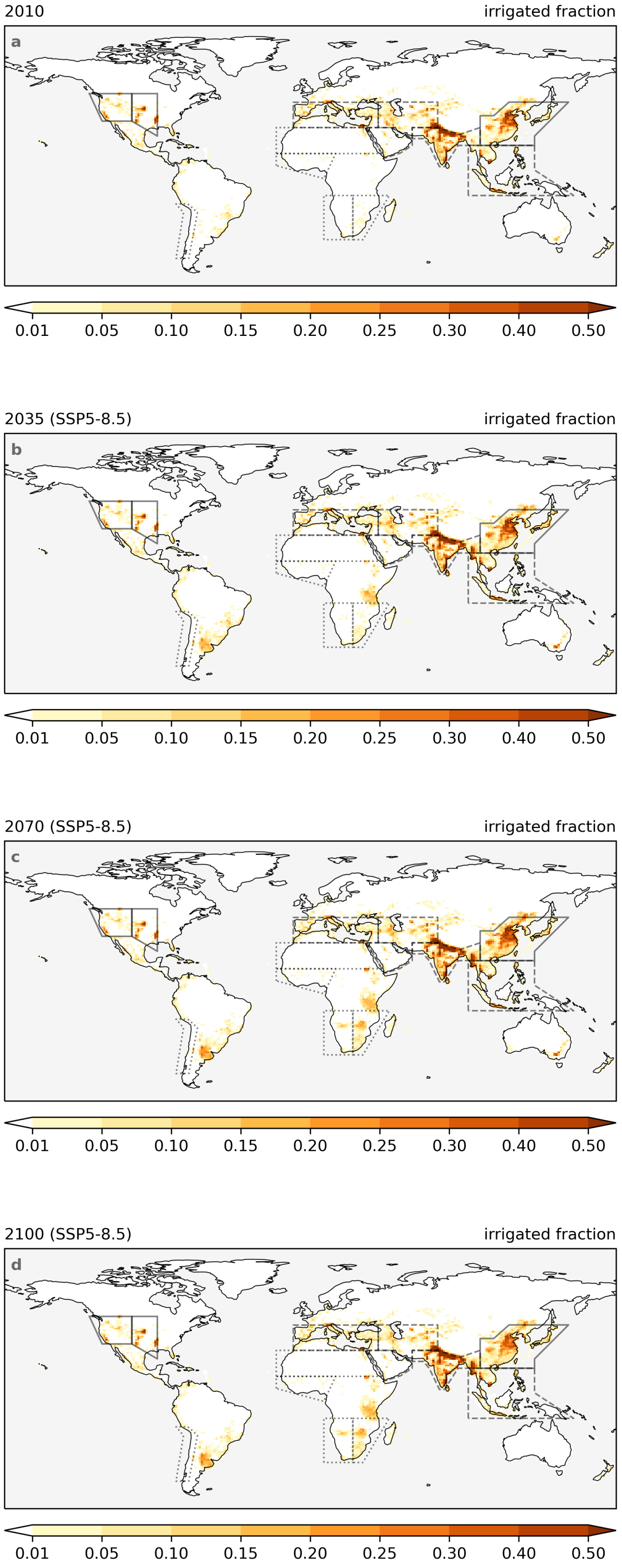

Supplementary Figure S9 | Irrigated fraction in the year 2010 (**a**), 2035 (**b**), 2070 (**c**), and 2100 (**d**), under SSP5-8.5. The spatial coverage of the IPCC reference regions[2] used in this study is indicated by solid lines, dashed lines, and dash-dotted lines.

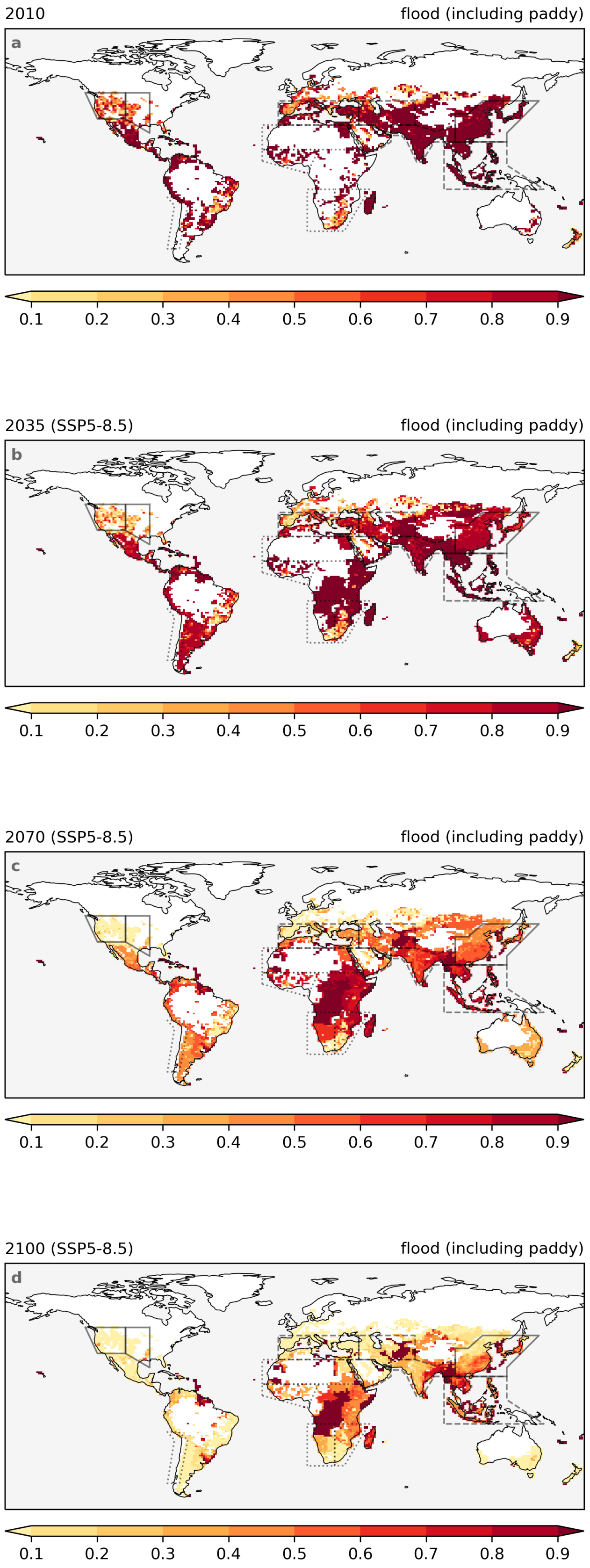

Supplementary Figure S10 | Fraction of flood irrigation in the year 2010 (**a**), 2035 (**b**), 2070 (**c**), and 2100 (**d**), under SSP5-8.5. The spatial coverage of the IPCC reference regions[2] used in this study is indicated by solid lines, dashed lines, and dash-dotted lines.

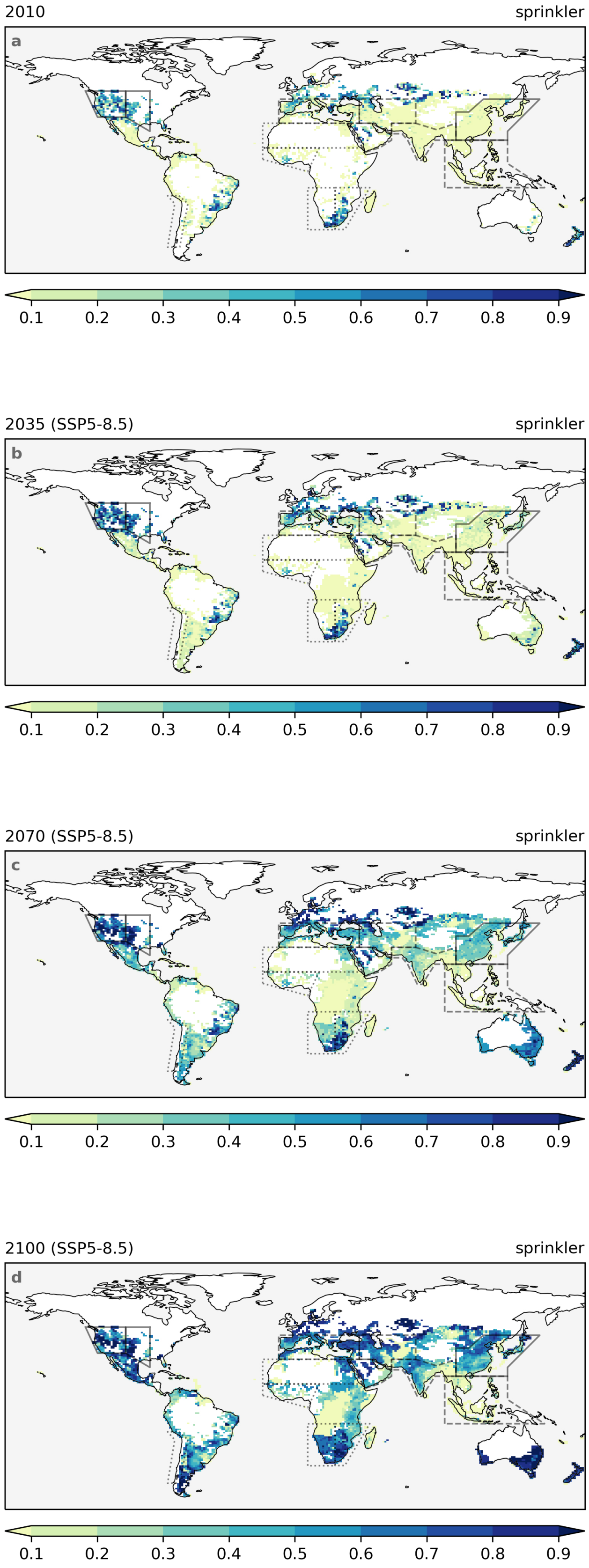

Supplementary Figure S11 | Fraction of sprinkler irrigation in the year 2010 (**a**), 2035 (**b**), 2070 (**c**), and 2100 (**d**), under SSP5-8.5. The spatial coverage of the IPCC reference regions[2] used in this study is indicated by solid lines, dashed lines, and dash-dotted lines.

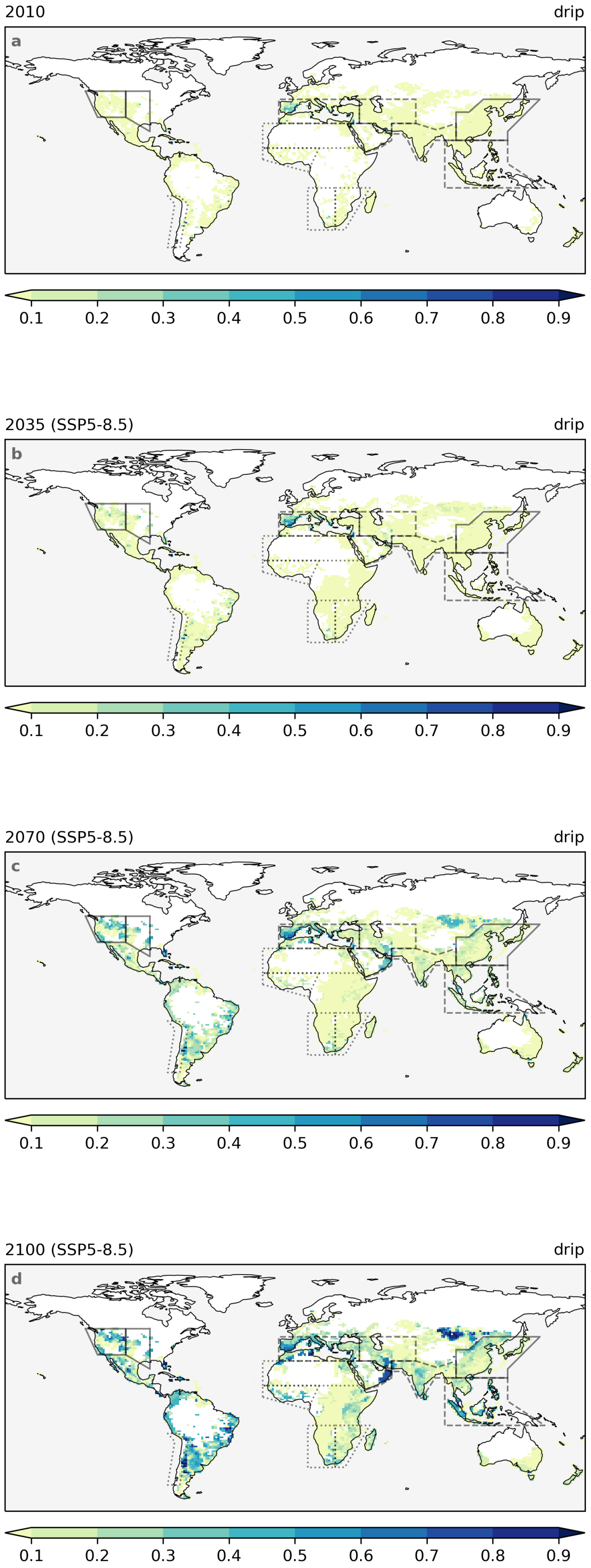

Supplementary Figure S12 | Fraction of drip irrigation in the year 2010 (**a**), 2035 (**b**), 2070 (**c**), and 2100 (**d**), under SSP5-8.5. The spatial coverage of the IPCC reference regions[2] used in this study is indicated by solid lines, dashed lines, and dash-dotted lines.

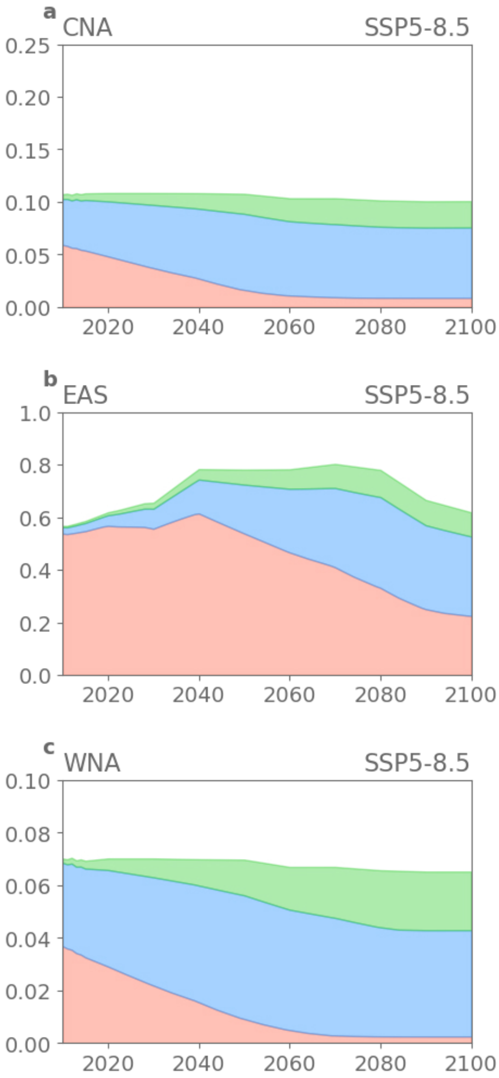

Supplementary Figure S13 | Group 1's regional areas equipped for irrigation (AEI) equipped with different irrigation techniques under SSP5-8.5. Regions include Central North America (**a**), East Asia (**b**), and West North America (**c**).

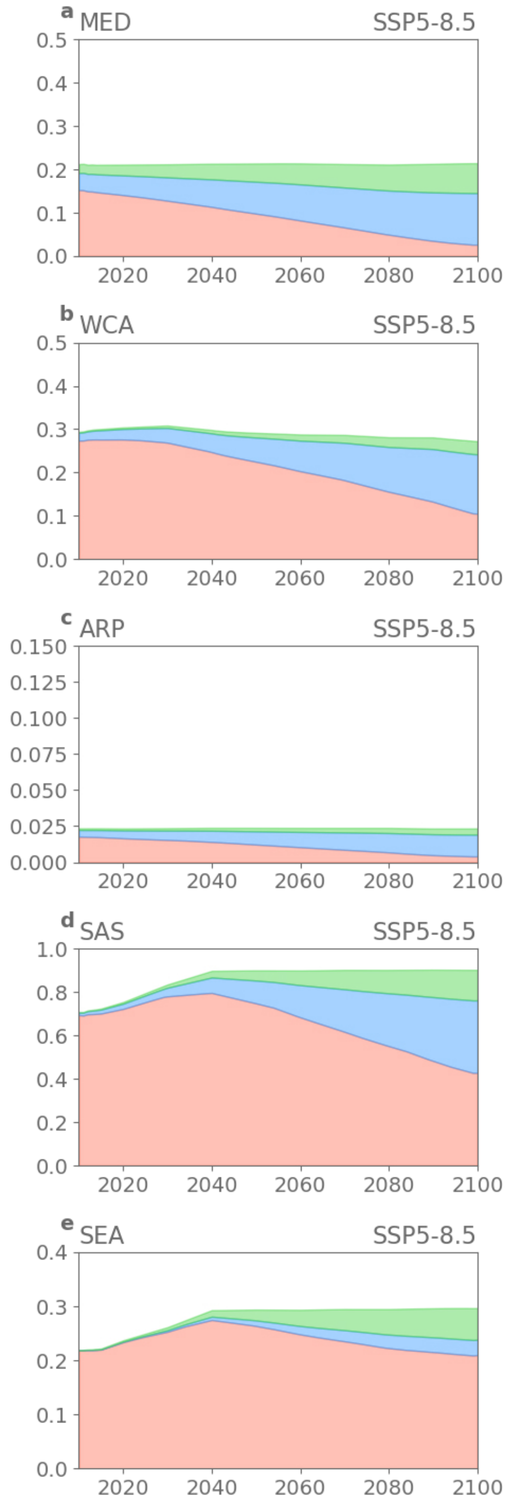

Supplementary Figure S14 | Group 2's regional areas equipped for irrigation (AEI) equipped with different irrigation techniques under SSP5-8.5. Regions include Mediterranean (**a**), West Central Asia (**b**), Arabian Peninsula (**c**), South Asia (**d**), and Southeast Asia (**e**).

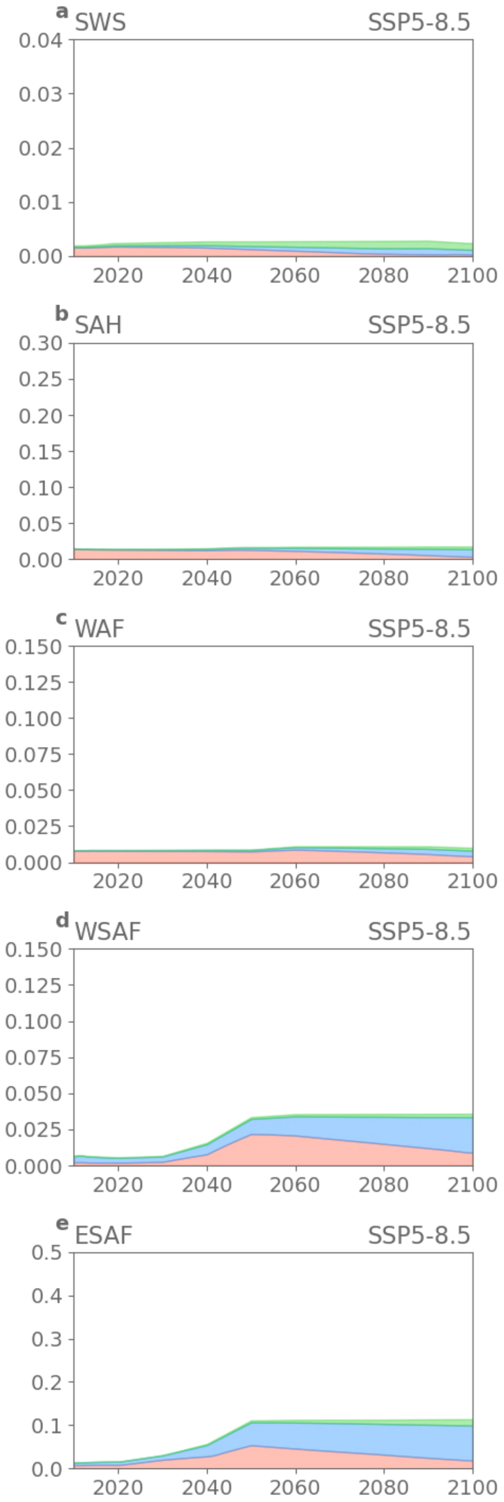

Supplementary Figure S15 | Group 3's regional areas equipped for irrigation (AEI) equipped with different irrigation techniques under SSP5-8.5. Regions include Southwest South America (**a**), Sahara (**b**), West Africa (**c**), West South Africa (**d**), and East South Africa (**e**).

Supplementary References

[1] Jägermeyr, J., Gerten, D., Heinke, J., Schaphoff, S., Kummu, M. & Lucht, W. Water savings potentials of irrigation systems: global simulation of processes and linkages. *Hydrology And Earth System Sciences.* **19**, 3073-3091 (2015)

[2] Iturbide, M., Gutiérrez, J., Alves, L., Bedia, J., Cerezo-Mota, R., Cimadevilla, E., Cofiño, A., Di Luca, A., Faria, S., Gorodetskaya, I. & Others An update of IPCC climate reference regions for subcontinental analysis of climate model data: definition and aggregated datasets. *Earth System Science Data.* **12**, 2959-2970 (2020)
